# Biologics in renal transplantation

**DOI:** 10.1007/s00467-014-2886-4

**Published:** 2014-07-26

**Authors:** Ryszard Grenda

**Affiliations:** Department of Nephrology & Kidney Transplantation, The Childrens Memorial Health Institute, Warsaw, Poland

**Keywords:** Biologic drugs, Renal transplantation, Mechanisms, Indications, Safety

## Abstract

The biologics used in transplantation clinical practice include several monoclonal and polyclonal antibodies aimed at specific cellular receptors. The effect of their mechanisms of action includes depleting or blocking specific cell subpopulations, complement system, or removing circulating preformed antibodies and blocking their production. They are used in induction, desensitization ABO-incompatible renal transplantation, rescue therapy of steroid-resistant acute rejection, treatment of posttransplant recurrence of primary disease such as nephrotic syndrome or atypical hemolytic–uremic syndrome, and in late humoral rejection. There are various indications for the use of biologic agents before and early or late after renal transplantation in both high- and low-risk recipients. In the latter situation, the biologics-based induction is used to further minimize immunosuppression maintenance. The targets of several biologic agents are present across a variety of cells, and manipulation of the immune system with biologics may be associated with significant risk of acute and late-onset adverse events; therefore, clinical risk-versus-benefit ratio must be carefully balanced in every case. Several trials on novel biologics are reported in adults but not in the pediatric population.

## Introduction

**Fig. 1:**
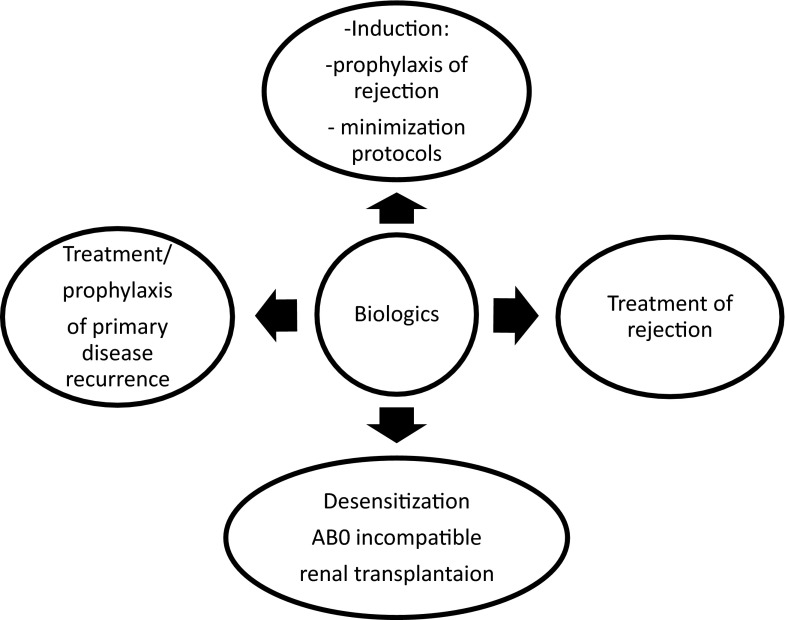
Indications to use biologics in renal transplantation

**Fig. 2:**
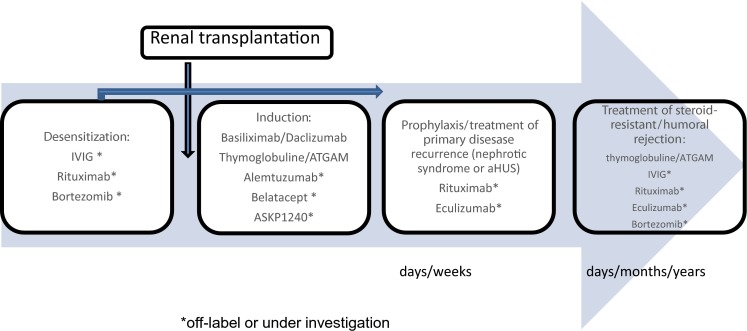
Timing biologic use in renal transplantation

Biologic agents used in renal transplantation include several drugs of different mechanisms of action, given intravenously, aimed at blocking or depleting specific cell subpopulations or blocking circulating alloantibody responses or the complement system. From a clinical standpoint, they are used in induction, desensitization procedures in hyperimmunized patients, ABO-incompatible renal transplantation, treatment of steroid-resistant and/or humoral rejection, and therapy for recurrence of specific primary renal disease, such as nephrotic syndrome (NS) or atypical hemolytic uremic syndrome (aHUS) (Fig. 1). They are used before and early or late after renal transplantation in both high- and low-risk recipients (Fig. 2). Several biologic agents are used in clinical practice in pediatric renal transplantation; however, the majority is used off-label, and their dose range and optimal number of doses are not clearly defined. This review summarizes data on experience, efficacy, and safety of biologics used in renal transplantation in children and on emerging new agents, used only in adult practice or clinical trials, that have not yet been verified in the pediatric setting. Drugs used in children include monoclonal antibodies (MAb) daclizumab, basiliximab, and alemtuzumab; polyclonal antibodies thymoglobulin or ATGAM [lymphocyte immune globulin, antithymocyte globulin (equine) sterile solution], specific MAb such as rituximab and eculizumab; and human immunoglobulin preparations [intravenous immunoglobulins (IVIG)]. Targets for and mechanisms of action of these agents are presented in Table 1. Indications, duration of action, and specific monitoring are presented in Table  2 [1–3, 11–19, 21–23, 25–28, 39, 42–47, 49, 53, 56, 57, 62–64]. A variety of new drugs were investigated in several clinical trials that recruited adult renal graft recipients [3, 4]. These new molecules were used to desensitize high-risk patients, in induction protocols, and to treat humoral rejection. Emerging new drugs and their specific targets in the immune system are listed in Table 3 [4]; clinical experience from clinical trials in adult patients is summarized in Table 4 [4–10], and evidence-based (EB) clinical experience in the pediatric population is presented in Table 5 [11–19, 21–23, 25–28, 33, 39, 42–47, 49, 53, 56, 57, 62–64].

**Table 1:** Biologic agents used in pediatric renal transplantation in clinical practice: targets in the immune system and mechanisms of action [1, 3]

Agent	Target	Suggested mechanism of action
Daclizumab (humanized MAb), basiliximab (chimeric MAb)	CD25 (IL-2 receptor α chain)	Binds to and blocks IL-2 receptor on T cells, inhibiting IL-2-induced T-cell activation
Alemtuzumab (humanized MAb)	CD52	Binds to CD52 receptor on T and B cells, monocytes, macrophages, and NK cells, resulting cell lysis and long-lasting depletion
Rituximab (chimeric MAb)	CD20	Binds to CD20 on B cells and mediates B-cell lysis and depletion
Eculizumab (recombinant humanized MAb)	Complement protein C5	Binds to complement protein C5, inhibiting its cleavage to C5a and C5b and preventing generation of terminal complement complex C5b-9
Thymoglobulin/ATGAM (polyclonal IgG)	T cells: CD3, CD4, CD8, CD58, CD28 and others B cells: CD5, CD58, CD28, CD152 and others APC: HLA-DR, CD58, CD80, CD86, CD40 and others Several receptors present on plasma cells, monocytes, dendritic cells, leucocytes, and others	Blocks several T- and B-cell receptors, causing cell dysfunction, lysis, and long-lasting depletion
IVIG (polyclonal human IgG)	Circulating alloantibodies and B cells	Related to antibody and B cells (selected mechanisms):
		Anti-idiotypic blockade of alloantibodies
		Downregulation of Ab production
		Increased catabolism of IgG caspase and mitochondrial-induced apoptosis of B cells

*MAb* monoclonal antibody,* ATGAM* lymphocyte immune globulin, antithymocyte globulin (equine) sterile solution,* IVIG* intravenous immunoglobulins,* IgG* immunoglobulin G,* IL-2* interleukin-2,* APC* antigen-presenting cells,* HLA* human leukocyte antigen,* NK* natural killer

**Table 2:** Biologic agents used in pediatric renal transplantation in clinical practice: indications, duration of effect, and monitoring

Drug	Group	Indications	Duration of effect on target cells	Monitoring	Number of doses
Basiliximab	MAb	induction	up to 56 days (with 2 doses)	Receptor CD20 saturation or drug concentration in serum (possible; not routinely used)	2
Daclizumab*	MAb	induction	number of doses, dependent effect	receptor CD20 saturation or drug concentration in serum (not routinely used)	2-6
Alemtuzumab***	MAb	Induction; treatment of rejection (not routine)	up to 12 months	CD52 count	1-2
Rituximab***	MAb	desensitization; refractory recurrence of nephrotic syndrome	up to 12 months (cumulative dose-dependent effect)	CD19 count	1-4
Eculizumab***	MAb	recurrence of aHUS; prophylaxis or treatment humoral rejection***	14 days (after single dose)	C5-dependent functional assay, TCC, CH50 in serum	1-**
Anti-T-cell Ab (thymoglobulin; ATG)	polyclonal ab	induction; steroid-resistant rejection	Up to 12 months (cumulative dose-dependent effect)	CD3 count WBC count	1-10
IVIG	Immunoglobulin	Desensitization	****	no	2-6

*ATG* antithymocyte globulin, * Ab* antibodies,* MAb* monoclonal antibodies,* aHUS* atypical hemolytic uremic syndrome, * IVIG* intravenous immunoglobulin G,* TTC* terminal complement complex,* CH50* 50 % hemolytic complement activity,* WBC* white blood cell

*not available since 2009

**undefined number of doses in prophylaxis of genetic HUS recurrence after renal transplantation

*** off-label in transplantation

**** duration of IVIG effect on circulating Ab is difficult to evaluate, as their further production is blocked by rituximab given simultaneously (in nonplasmapheresis protocols); significant decrease in anti-HLA Ab class II titer as early as from days 10–20 after first dose of IVIG was reported [35]

**Table 3:** New investigational agents (not used in pediatric clinical practice or evaluated in pediatric clinical trials)

Agent	Target	Suggested mechanism of action
Belatacept	CD80 (B7-1), CD86 (B7-2) on APCs	Blockade of pathway preventing production of IL-2 and conversion of naïve T cells into effector T cells
Alefacept*	CD2 on T cells	Co-stimulation inhibitor. Preferential depletion of effector T cells
Natalizumab	VLA-4 on lymphocytes	Blockade of interaction between VLA-4 and VCAM-1 and impairment of lymphocyte trafficking into tissues
Efalizumab*	LFA-1 (CD11a) on leukocytes	Competitive inhibition with ICAM-1 located on APCs and impairment of lymphocyte adhesion and activation
Tasocitinib (tofacitinib)	JAK3 (JANUS tyrosine kinase)	Inhibiting signaling cascade by blocking transcriptive factors Stat 5a i Stat 5b
Bortezomib	Proteasome	Inhibiting degradation of cell-cycle regulatory proteins resulting in cell-cycle arrest and apoptosis; inhibiting degradation of inhibitor κB, therefore preventing NFκB-mediated cell activation
ASKP1240	CD40	Blockade of CD40-positive cells

*ASKP1240* a fully human anti-CD40 monoclonal antibody, *APC* antigen-presenting cell,

*IL*-2 interleukin-2

*ICAM*-1 intracellular adhesion-cell-molecule-1

*LFA*-1 lymphocyte-function-associated antigen

*VCAM*-1 vascular adhesion-cell-molecule-1

*VLA*-4 very late antigen-4,* NFκB* nuclear factor kappa B

*No further investigation in transplantation

**Table 4:** Clinical experience with novel drugs (still being investigated) in adult transplant populations

Agent	Major reports	Clinical indication; treated populations	Efficacy; other benefits	Safety; specific caution
Belatacept	RCTs: BENEFIT study BENEFIT EXT study	Induction: adults (*n* = 445; in two treatment arms); adults (*n* = 102)	Not inferior to CNI-based triple regimen. Better metabolic profile compared to CNI	High risk of PTLD (CNS specific); strongly contraindicated in EBV-naïve patients
Tasocitinib (tofacitinib)	RCT	Induction: adults (*n* = 40, in two treatment arms)	Not inferior to CNI-based triple regimen	High rate of viral infection Dyslipidemia
ASKP1240	RCT	Induction: overall 38 (3 treatment arms)	Not inferior to CNI-based triple regimen	Significant rate of infections
Bortezomib	Case series	Desensitization: treatment of antibody-mediated rejection; largest series* n* = 70)	Promising	Anemia; peripheral neuropathy

*RCT* randomized controlled trial *CNS* central nervous system, *CNI* calcineurine inhibitor, *PTLD* posttransplant lymphoproliferative disease,* EBV* Epstein–Barr virus

**Table 5:** Evidence-based medicine level of clinical experience with biologics in pediatric renal transplantation

Indication	Agents	Type of report	Overall number of treated patients*	Overall outcome
Induction	Anti-CD25 Ab; anti-CD52 Ab; polyclonal Ab	International and national multicenter RCTs: single-center prospective trials, single-center case–control studies	1,503	Favorable graft and patient survival; clinical benefit from steroid or/and CNI minimization (in relevant studies); satisfactory safety profile
Desensitization; HLA incompatible ABO incompatible	IVIG, rituximab	Case series and case reports	65	Transplantation possible; shorter waiting time; no proven long-term efficacy. Transplantation possible; favorable long-term patient and graft survival
Treatment of primary disease recurrence	Rituximab, eculizumab	Case series and case reports	38	Variable efficacy in NS. Effective prophylaxis in most cases of aHUS. Effective in treatment of aHUS recurrence
Treatment of steroid resistant rejection	Polyclonal Ab: rituximab, alemtuzumab	Single-center prospective trials; case series and case reports	27	Variable efficacy in acute rejection
Treatment of chronic humoral rejection	IVIG, rituximab	Single-center prospective trials; case series and case reports	26	Effective in majority (70 %) of reported patients

*HLA* human leukocyte antigen, AB antibodies, *IVIG* intravenous immunoglobulin, *RCT* randomized controlled trials, *CNI* calcinurine inhibitors,* NS* nephrotic syndrome,* aHUS* atypical hemolytic uremic syndrome

*Overall number of patients means the sum of cases presented in quoted publications listed in the text, not the overall number of patients ever treated with a particular drug

## Biologics in induction

Induction is the most common indication to use biologic agents in pediatric renal graft recipients. It is used for two reasons: (1) to enhance the strength of initial triple immunosuppression in patients with high immunological risk [sensitized, retransplanted, poor human leukocyte antigen (HLA) matching or marginal donor transplant] or (2) to introduce minimization protocol aimed at reducing exposure to steroids or calcineurin inhibitors (CNI), or both. In the second indication, MAbs were used in patients with low immunological risk and polyclonal Ab in patients with low and high immunological risk.

MAb used in pediatric transplantation include anti-CD25 (IL-2Rα) inhibitors daclizumab and basiliximab and anti-CD52-depleting Ab alemtuzumab used off label. The duration of effect (for two doses of basiliximab) expressed as receptor saturation was present at about 5 weeks with no mycophenolate mofetil (MMF) and about 10 weeks with concomitant MMF therapy [11]. In the Stanford steroid avoidance trial, the trough concentration of daclizumab was monitored by sequential sandwich enzyme-linked immunosorbent assay (ELISA); however, routinely, no specific monitoring is used in practice [12]. Comprehensive information on efficacy and safety of anti-CD25 inhibitors basiliximab and daclizumab, including in renal-graft recipients of different ages, comes from a Cochrane database large systematic review involving 71 adult and pediatric trials and 10,520 participants. Use of both daclizumab and basiliximab given in induction decreased the risk of acute rejection in the first year after transplantation by 25 % [relative risk (RR) 0.75], as well as incidence of 1-year graft loss by 25 % [13]. Two pediatric randomized controlled trials (RCTs) proved that adding anti-CD25 Ab basiliximab to triple-maintenance protocol tacrolimus/azathioprine/prednisolone (TAC/AZA/Pred) or cyclosporin A/MMF/Pred (CsA/MMF/Pred) in patients of low to moderate immunological risk is not justified, as the incidence of rejection and patient and graft survival was no different in children with or without induction [14, 15]. Monoclonal induction was also used in a majority of pediatric clinical trials on steroid minimization. The Stanford complete steroid avoidance study was based on an extended daclizumab induction (overall nine doses) [12]. Only two daclizumab doses of 1 mg/kg were used in the TWIST trial, and steroids were stopped at day 5 after surgery [16]. It should be noted that daclizumab is no longer manufactured, and two doses of basiliximab were used in further pediatric trials on steroid withdrawal [17–19]. Early and long-term results of all these and other trials have shown that in pediatric patients with low to moderate immunological risk, monoclonal induction with anti-CD25 Ab with combination TAC/MMF therapy was sufficient to allow early steroid withdrawal, resulting in all expected clinical benefits, including better growth, with no detrimental effect on long-term patient/graft survival and renal function [20]. Basiliximab was also used in the innovative protocol, which aimed to double the minimization of immunosuppression (CNI plus steroids). With monoclonal induction and use of everolimus, reduced exposure to cyclosporine was possible; in further follow-up, with a normal renal biopsy, the late (> 6 months after transplantation) steroid withdrawal was also possible. This protocol was very effective in patients at low immunological risk, with no rejection within 1 year and with 100 % patient and graft survival in the 3-year follow-up [21]. The ongoing multicenter CRADLE RCT aims at verifying the efficacy and safety of a similar protocol but with Csa replaced by TAC, especially in the subgroup of patients given basiliximab, as monoclonal induction with basiliximab is not mandatory in this trial and depends on the individual decisions of each center (www.clinicaltrials.gov/ct2/show/NCT01544491).

Induction with basiliximab and the then new drug belatacept, administered IV every 2 weeks then repeated infusions every 4 weeks, was used for prophylaxis of acute rejection in adult patients after renal transplantation, randomized to three arms, including two with different dosages of belatacept and one with CsA, all combined with MMF and steroids. At 1 year, both belatacept arms showed no inferiority to the CsA arm in terms of graft and patient survival, with better renal function in belatacept arms. An important clinical benefit was better metabolic profile in belatacept-treated patients; however, there was a safety concern related to high posttransplant lymphoproliferative disease (PTLD) rate in Epstein-Barr virus (EBV)-seronegative patients [5]. Another option of monoclonal antibody induction was the use of alemtuzumab, primarily in children, by Pittsburgh group, who used the single dose of 0.4–0.5 mg/kg, followed by TAC monotherapy and early steroid withdrawal (at 1–5 days after transplantation). Using this approach and long-lasting depletion of target cells, maintenance immunosuppression was limited to TAC monotherapy [22, 23]. 

An innovative protocol of alemtuzumab induction (30 mg/dose) with monthly belatacept IV (10 mg/kg/dose) and daily sirolimus given after renal transplantation to avoid calcineurin and steroid exposure in 20 adult renal transplant recipients was recently reported. There was no acute rejection or de novo donor-specific antibody (DSA) production within the first year. Ten patients remained on belatacept as the single immunosuppressive drug [24].

### Polyclonal induction

In a retrospective single-center study, 198 children and adolescents were given polyclonal combined with triple-maintenance regimen. Significantly fewer episodes of acute rejection were seen in patients treated with thymoglobulin (33 % vs 50 %, *p* = 0.02) [25]. Overall, 37 adolescents (mean age 15.2 ± 2.8 years) were treated with the induction protocol, including five to seven fixed doses of 1.5 mg/kg thymoglobulin combined with TAC/MMF/Pred; there was an 8.1 % incidence of acute rejection within 1 year and 91.9 % graft and 100 % patient survival [26]. Six fixed doses of 1.5 mg/kg thymoglobulin induction were used by the Stanford group in an early steroid withdrawal protocol in 13 children with high immunological risk, with no further rejection episode within 1-year follow-up, normal picture biopsies performed every 3 months up to 1 year after transplantation, and no de novo DSA [27]. Overall, five to seven doses of 1.5 mg/kg thymoglobulin were given to 21 pediatric patients undergoing an early steroid minimization protocol compared with six to 15 doses given as steroid maintenance (retrospective control group). With steroid withdrawal on day 6, the incidence of acute rejection was 23 % and graft survival 90 % at 1 year, which was no different than in controls [28]. 

The optimal number of thymoglobulin doses (days of treatment) in induction protocols is not defined. The attempt to keep optimal balance between efficacy and safety is the basis of using short (<3 doses) or longer (up to 10 doses) induction and adjusting the dose to trough CD3 (target 50–100/mm^3^) or WBC count (target > 3000/mm^3^) versus a fixed dose of 1.5 mg/kg [29, 30]. The median cumulative dose in adult renal graft recipients in TAILOR registry data was 5 mg/kg per treatment (1.56-15.00); 46.6 % of patients (overall *n* = 2,322) received between 1.5 and 5 mg/kg, 35.4 % from 5 to 7 mg/kg, and 18 % from 7 to 15 mg/kg per treatment. Up to 64.6 % of patients tolerated the full intended induction dose [31]. The US Organ Procurement and Transplantation Network (OPTN) database stratified the incidence of based on depleting and nondepleting agents: lymphocyte-depleting Ab were used in 47.5 % and IL-2R antibodies in 43 % of 1,276 children treated with steroid minimization protocols and induction between 2002 and 2009 [32]. 

Summarizing: induction with biologic agents, such as anti- IL2 Ab (basiliximab) or polyclonal Ab, after verification in clinical trials and reports, has entered routine practice in selected patients with clear clinical indications.

## Biologics in desensitization of HLA-incompatible renal-graft recipients

In patients awaiting renal transplantation, reducing the titer of pre-formed anti-HLA antibodies by using plasmapheresis (PF), IVIG administration alone, combined with PF, or rituximab was reported in several adult studies and a few pediatric case reports [33–35]. The most common protocol used in adult patients in the USA was based on a combination of IVIG and PF (82 %), and preoperative rituximab was used in more than half of reporting centers [36]. The combination of IVIG and rituximab was used by Jordan’s group to reduce the titer of pre-formed anti-HLA antibodies in highly sensitized patients awaiting renal transplantation [37]. The authors described the protocol based on administration of 2 g/kg IVIG on the days 0 and 30, combined with rituximab (375 mg/m^2^) given on days 7 and 22 (after first dose if IVIG). This protocol caused significant reduction of mean panel-reactive antibody (PRA) level from 77 ± 19 % to 44 ± 30 % (*p* < 0.0001) after second infusion of IVIG and shortened the waiting time to successful transplantation from 144 ± 89 months to 5 ± 6 months. Regardless of the encouraging short-term results, the long–term efficacy of the desensitization protocol was questioned in adult patients in a retrospective comparative cohort study. The 1- and 5-year graft survival rate was significantly inferior in patients who underwent the desensitization protocol based on a course of PF combined with IVIG and then depletional induction (89.9 vs 97.6 % and 69.4 vs 80.6 %, respectively). The overall risk of graft loss was significantly higher in desensitized patients [hazard ratio (HR) 2.6; *p* = 0.04)] but with no detrimental effect on patient survival [38].

## Biologics in ABO-incompatible renal transplantation

Other specific desensitization protocol was used by Tyden et al. in living- donor ABO-incompatible pediatric transplantation. The pretransplant protocol included a single dose of 375 mg/m^2^ rituximab given 4 weeks before scheduled immunoadsorption, triple immunosuppression, four sessions of antigen-specific immunoadsorption, followed by 0.5 g/kg IVIG preoperatively and continuation of immunoadsorption after surgery. Five-year patients survival rate was 98 % and graft survival rate 97 % [39], which are obviously not inferior to outcomes in ABO-compatible transplantation. Eculizumab was used to enhance the desensitization protocol (PF followed by polyclonal induction) in adult patients undergoing living-donor renal transplantation against positive cross match in terms of preventing antibody-mediated rejection (AMR). Overall, 26 patients received preemptively 1,200 mg of eculizumab immediately before transplantation, then 600 mg on day 1, then four times weekly. Further dosing was adjusted to the presence of DSA. The incidence of antibody-mediated rejection (AMR) was 7.7 % vs 41.2 % in the control group (*p* = 0.0031) [40]. Bortezomib (4 x 1.3 g/m^2^) was given pre-emptively (early posttransplant) to remove de novo DSA (before expected further humoral rejection occurs). In a series of 26 patients, bortezomib combined with steroids was given within a mean of 30 days after DSA appearance (*n* = 26), with PF and rituximab (*n* = 9), with PF only (*n* = 5), or with IVIG (*n* = 1). There was significant reduction in DSA level at 1 year (*p* = 0.002), correlated with better allograft function at a mean of 25.8 months of follow-up [41]. 

Summarizing: Desensitization of HLA-incompatible patients with biologic agents (or combination of biologics with plasma exchange) allows further renal transplantation and may shorten the waiting time for transplant. However, these patients still present a high risk of rejection and inferior long-term graft survival. This will be very important in pediatric renal-graft recipients, who have a long life expectancy on renal replacement therapy. A specific desensitization protocol, as described by Tyden et al., allows successful ABO-incompatible renal transplantation with excellent long-term outcome.

## Biologics in recurrence of primary disease after renal transplantation

Eculizumab has been used in prophylaxis and treatment of posttransplant recurrence of aHUS. The report by the French Group for Atypical HUS assessed 22 pediatric cases in their retrospective multicenter study. Thirteen patients were treated due to recurrence, and nine received pre-emptive eculizumab as prophylaxis. Single and multiple doses were given is cases presenting several* CFH*,* CFI*, and* C3* gene mutations. Some patients were resistant to PF (*n* = 10), and some were PF dependent (*n* = 2). In some cases, the picture of renal biopsy included signs of acute rejection. All but one patient from the prophylaxis group remained recurrence free at a mean 14.5-month follow-up. In all 13 patients with recurrence, the hematological features of aHUS rapidly returned to normal following eculizumab administration, whereas mean creatinine concentration dropped from a mean of 295 ± 171 to 135 ± 69 μmol/L (*p* = 0.002) within 3 subsequent months. Patients with delayed introduction of eculizumab treatment (>28 days after diagnosis) had lower functional benefit than patients treated earlier after aHUS onset [42]. Rituximab was used to treat recurrence of severe NS in a series of children after renal transplantation. One to four doses (of 375 mg/m^2^) were given, and complete or partial response was observed in six of seven patients [43]. Variable response to one to four doses of rituximab was reported in series of eight patients; complete response was seen in two and partial in four. In some cases, there was a correlation between CD19 depletion and clinical response and between CD19 recovery and relapses; in others, there was no association between CD19 count and clinical course of NS [44]. The efficacy of rituximab (four doses) in a girl with Finnish-type congenital NS and clinical “recurrence” due to anti-nephrin Ab production was reported. Remission was sustained during the 5-year follow-up [45].

 Summarizing: recurrence of severe primary renal diseases has become potentially treatable with currently available biologic agents (rituximab and eculizumab); however, important limitations remain, including overall efficacy and safety for rituximab and enormous financial cost for eculizumab in prolonged prophylaxis.

## Biologics in rejection therapy

### Acute rejection

Acute rejection is associated with allograft infiltration by several cell types, including T and B cells, macrophages, and NK cells. Polyclonal Ab therapy is commonly used in steroid-resistant cases. The significant presence of B cells in biopsy-proven infiltrate may suggest that rituximab might be useful. The Stanford group conducted a randomized trial comparing 4 weekly doses of rituximab (375 mg/m^2^) versus thymoglobulin (6 x 1.5 mg/kg) in 20 pediatric renal recipients with late acute rejection (mean time from transplantation to rejection 34 and 21.36 months) in two arms of ten patients each. All patients were pretreated with methylprednisolone (MP). . In six patients in the rituximab group and two from the control group, humoral component of rejection was confirmed by CD4 presence. The presence of CD20 cells in graft infiltrates was confirmed in all patients. Results confirmed the efficacy of rituximab therapy in CD20-positive acute rejection [46]. Limited efficacy of single-dose (0.3 mg/kg) alemtuzumab in rescue treatment of late acute cellular rejection was reported in three children at high immunological risk, with recurrent episodes of rejection and poor previous response to other therapies (including steroids and polyclonal Ab) [47]. More successful treatment was described in 15 adult patients. The use of multiple doses (4–10 days, dose 6–10 mg/kg) showed no increase in malignancy or cytomegalovirus (CMV) infection over 10 years of follow-up despite, high doses used in this study [48]. Whether or not this difference in efficacy was related to the use of single versus multiple doses, rejection type, or patient specificity is not clear. Three successful pediatric cases of ABR were reported with the use of MP, 2 g/kg IVIG, a single dose of rituximab (375 mg/m^2^), and course of plasma exchange (up to ten procedures) [49]. The protocol proposed by Jordan et al., based on clinical experience, distinguished patients with less and more pronounced pathologic features of ABR. Those with a milder clinical picture received a combination of three MP pulses, initial dose of 2 g/kg IVIG given between first and second pulses, and a single dose of rituximab (375 mg/m^2^) on day 2, followed by two consecutive MP pulses. The second (and last) dose of 2 g/kg IVIG was given between 30 and 60 days of treatment. More severe cases, with signs of thrombotic microangiopathy in biopsy, were treated with a series of PF, followed by single doses of IVIG (2 g/kg) and a single dose of rituximab (375 mg/m^2^) [50]. Bortezomib, in a retrospective comparison with rituximab, was reported in ten adult patients treated also with MP, PF (six sessions), and IVIG (30 g/treatment) [51]. The efficacy of bortezomib was lower in cases of late AMR, i.e., occurring > 6 months after transplantation. Delay in diagnosis and difference in characteristics of cells producing DSA in late-onset AMR (long-lived plasma-cell population) is proposed as an explanation of a worse response [52]. Eculizumab was successfully used in a hyperimmunized 17-year-old patient with PF resistant of acute humoral rejection, developing in the second graft, after a desensitization protocol based on combined PF, IVIG, and rituximab. The patient was treated with MP, repeated PF, and IVIG; however, DSA and ongoing rejection persisted, proven in repeated biopsy, despite 45 sessions of PF and absence of CD19 cells. Four doses of eculizumab (600 mg) were given, and remission was present at the third biopsy. Eculizumab was then continued on a monthly basis (eight doses) due to increasing DSA titer, which appeared after PF was stopped. Two years after therapy, the patient was stable, with creatinine concentration of 1.2 mg/dl and DQ DSA of mean fluorescent intensity (MFI) 5,000 [53]. Similar reports were published regarding eculizumab efficacy in highly sensitized adults patients developing acute humoral rejection despite pretransplant (PF/IVIG/rituximab) desensitization and use of polyclonal induction; one to five doses of eculizumab were used in rescue therapy [54, 55].

### Chronic humoral rejection

The protocol described by Jordan et al. in patients with chronic antibody-mediated rejection as a result of the presence of de novo DSA had three options: IVIG alone, IVIG plus rituximab, and combination of plasmapheresis with lower dose of IVIG (1 g/kg) with or without rituximab [50]. The combination of IVIG (4 × 1 g/kg) and a single dose of rituximab (375 mg/m^2^) given 1 week after last IVIG dose was reported as a therapeutic tool for chronic humoral rejection in six children: four responded to antirejection therapy and showed increased glomerular filtration rate (GFR) within 12 months: significantly at 6 months by 21 ml/min/1.73 m^2^ (*p* < 0.05) and then not significantly at 12 months by 19 ml/min/1.73 m^2^ (*p* = 0.063) [56]. The same protocol (four doses of IVIG 1 g/kg plus a single dose of 375 mg/m^2^ rituximab) was used in a prospective study recruiting 20 children with Banff diagnostic criteria of Ab-mediated rejection together with CD20-positive infiltrates (12 (60 %) patients). The response rate was 70 % (14 patients) [57]. Bortezomib (4 × 1.3 g/m*2*) was used in adult patients with humoral rejection as a single rescue agent or in combination with IVIG or PF [58]. 

Summarizing: Availability of biologic agents increases the range of therapeutic tools in resistant, acute, and humoral chronic allograft rejection. However, selecting the potentially most effective treatment protocol requires very detailed diagnosis based on close DSA monitoring and interpretation of results and relevant pathomorphologic evaluation, including the phenotype of infiltrating cells (T or B). There remains a need for further clinical investigation in this area.

## Safety concerns with biologic agents in clinical practice

Biologic agents used in renal transplantation are highly potent immunosuppressants, interfering with normal immune response and therefore increasing the risk of specific complications. Their safety profile is variable and might be dose dependent and related to cumulative side effects of certain drugs combinations. The safety profile of commonly used biologics is summarized in Table 6 [59–66].

**Table 6:** Safety profile of biologics used in pediatric renal transplantation

Event	Anti-IL-2R (basiliximab, daclizumab)	Polyclonal Ab (ATG)	Alemtuzumab	Rituximab	Eculizumab	IVIG
Cytokine release syndrome (fever, chills, hypotension)	0/+	+++	+	0/+	0	0
Hypersensitivity reactions	++	+++	+	+	+	+
Bone marrow complications	+	+++	+	+	+	0
Lymphopenia	+	+++	+++	+++	0	0
Malignances	0	++	+	0	0	0
Viral infections (CMV, EBV, BKV, other)	+	+++	++	+	++	+
Bacterial and fungal infections	+	++	++	0/+	0/+	+
Specific antibody formation	+	++	0	0	0	0
Other (specific)				RALI	Higher risk of meningitis (vaccination mandatory)	Transient acute kidney injury

*CMV* cytomegalovirus,* EBV* Epstein-Barr virus,* BKV* BK virus,* IL-2* interleukin 2,* Ab* antibodies* ATG* antithymoglobulin,* RALI* rituximab-associated lung injury,* IVIG* intravenous immunoglobulin,

### Posttransplant lymphoproliferative disease

The risk of posttransplant lymphoproliferative disease (PTLD) in pediatric renal graft recipients is higher than in adults mainly due to higher incidence of EBV seronegativity in the first decade of life, and depletional induction is regarded as an additional risk factor. The incidence of PTLD was significantly higher in children < 10 years of age treated with polyclonal Ab [59, 60]. Analysis of PTLD risk relation used various antibodies in 59,560 kidney recipients [data from the Organ Procurement and Transplantation Network/United Network for Organ Sharing (OPTN/UNOS)], including 3,105 children, showed that only polyclonal induction was associated with significant risk (RR 1.63, *p* = 0.0025 vs no induction). Additional factors included age <18 years (RR 3.67; *p* < 0.0001), seronegative EBV (RR 5.225; *p* < 0.0001), CMV (RR 2.036; *p* < 0.0001) status, and—interestingly— the use of sirolimus in maintenance immunosuppression (RR 2.047; *p* < 0.0001) [61]. A more recent report provides data on a higher risk of PTLD in patients receiving alemtuzumab for induction [62]. Pretransplant EBV seronegative status was also a risk factor of PTLD in children given nondepletional induction with basiliximab combined with sirolimus, tacrolimus, or cyclosporine, and steroids. Up to 6.9 % of patients developed PTLD. This was mainly seen in young EBV-naïve children receiving an EBV-seropositive renal allograft [18]. Of note is the specific relation of the new drug belatacept to PTLD (in adult patients): in EBV-seronegative cases, treatment was associated with a high incidence of PTLD, affecting in particular the central nervous system [5]. As the incidence of EBV-seronegative status is much higher in the first decade of life [59], the safety of further use of belatacept in pediatric patients is questionable.

### Infections

As reported by North American Pediatric Renal Trials and Collaborative Studies (NAPRTCS), induction is associated with significant higher risk of infection [odds ratio (OR) 1.45;* p* < 0.001), especially in terms of viral etiology (OR 1.47; *p* = 0.003) and in young children < 2 years of age, including the risk of BK–JC polyoma-virus (JVC)-related nephritis. The incidence of hospitalization within 2 years of follow-up (after induction) due to viral infections was about 30 % and bacterial infections about 28.4 % [63, 64]. Specific vaccination is mandatory with the use of eculizumab to prevent meningitis [42]. The association between treatment with rituximab JVC and, in consequence, development of progressive multifocal leukoencephalopathy (PML) was reported mainly in bone marrow transplant patients. The relevant report described 5.5 % incidence of JVC replication in adult solid-organ recipients (in the majority renal transplant patients) treated with rituximab, suggesting the need for close monitoring [65].

### Rituximab-specific serious adverse event: rituximab-associated lung injury

Rituximab-associated lung injury was reported in patients treated with anti-CD20 Ab. This life-threatening syndrome may include interstitial pneumonitis, alveolar–interstitial pneumonia, and rapidly progressing pulmonary fibrosis. The need for mechanical ventilation is a predictor of poor prognosis [66].

Summarizing: Use of biologic agents is associated with risk of several general or drug-specific adverse events; therefore, the risk/potential clinical benefit ratio must be carefully balanced. EBV-seronegative status, the most common in children < 10 years of age, is a specific problem in the pediatric population and may limit or increase the risk of biologic agent use.

**Key points regarding the use of biologics in rejection therapy are**:

- Biologics are used in transplantation to remove circulating antibodies and block their production, to reduce further exposure to toxic maintenance immunosuppressives, to treat recurrence of specific primary disease, and to treat severe rejection.
- Depending on indication, biologics may be used before and early and late after renal transplantation.
- Clinically additive mechanisms of action promote a combination of these drugs in specific clinical situations, such as desensitization or humoral rejection.
- Biologic targeting of receptors on different cells may enhance the risk of serious adverse events, despite specific prophylactic measures.

**Research points in biologics use in rejection therapy are**:

- Optimal treatment protocol for chronic humoral rejection should be established in controlled trials.
- Optimal combinations of biologic agents (and/or extracorporeal procedures) aimed to reduce circulating DSA level for prophylaxis of humoral rejection need to be established in sensitized candidates for transplantation and active post-transplant de novo DSA producers Optimal treatment of primary disease recurrence after transplantation, including rituximab for NS and eculizumab for aHUS, should be verified in controlled trials.
